# Stigma and Human Rights Abuses against People Who Inject Drugs in Russia—A Qualitative Investigation to Inform Policy and Public Health Strategies

**DOI:** 10.1371/journal.pone.0136030

**Published:** 2015-08-25

**Authors:** Karsten Lunze, Fatima I. Lunze, Anita Raj, Jeffrey H. Samet

**Affiliations:** 1 Boston University School of Medicine, Boston, MA, United States of America; 2 Boston Children's Hospital, Harvard Medical School, Boston, MA, United States of America; 3 Division of Global Public Health, Department of Medicine, University of California at San Diego School of Medicine, San Diego, CA, United States of America; University of North Carolina at Chapel Hill, UNITED STATES

## Abstract

**Introduction:**

Drug policing practices in the Russian Federation (Russia) are often punitive and have been shown to be associated with HIV risk behaviors among people who inject drugs (PWID). Less is known about strategies to address the problem in that setting, where substance use stigma is highly persistent. A better understanding of forms, causes and consequences of drug policing in Russia could inform drug policy in a context of substantial policy resistance. This qualitative study’s goal is to characterize the phenomenon of police involvement with Russian PWID and to explore strategies for drug policing in the Russian country context.

**Methods:**

Using a semi-structured interview guide, we collected data from a purposive sample of 23 key informants including PWID, police officers, and experts from civil society and international organizations in Russia. We used a thematic analysis approach to inductively generate new insight into the phenomenon of police involvement and potential strategies to address it.

**Results:**

Policing practices involving PWID include unjustified arrests, planting of false evidence and extrajudicial syringe confiscations, and often constitute human rights violations. Russian PWID personally experienced police violence as ubiquitous, taking on various forms such as beating, unjustified arrests, verbal harassment, and coercion. The persistent societal stigma dehumanizes PWID, and such stigmatization facilitates police abuse. To address stigma and overcome the PWID-police adversity, study participants suggested fostering a mutual understanding between the police and public health sectors.

**Conclusions:**

Participants describe substantial human rights violations as part of policing illicit drug use in Russia. Police should include principles of effective prevention of substance use and HIV risk reduction in their trainings. Alignment of public safety and public health goals could address drug use-related risks and HIV prevention among key populations in Russia.

## Introduction

Mas é tão consoladora

A vaga e triste canção.

Que a minha alma já não chora

Nem eu tenho coração…

Sou uma emoção estrangeira,

Um erro de sonho ido.

Canto de qualquer maneira

E acabo com um sentido!

Fernando Pessoa, Poesias Inéditas (1919–1935), 1^a^ ed. 1956

In the Russian Federation (Russia), injection drug use is the primary route of transmission of the HIV epidemic, which is at risk of bridging from high-risk groups to the general population [1]. Injection drug use has been increasing since the break-up of the Soviet Union and the subsequent political and socioeconomic turbulence [2]. In parallel, the country has experienced a dramatic increase in HIV prevalence from less than a thousand transfusion-related cases in the early 1990s to close to a million people currently infected [3].

The majority (64–82%) of Russians infected with HIV are PWID [4], among whom transmission occurs through unsafe injection behaviors such as sharing of needles and drug paraphernalia [5]. Such HIV epidemics concentrated in key populations carry the potential of viral spread to their sexual partners, the so-called “bridge populations.” Unsafe sex behaviors among PWID and their non-drug-using sex partners are common [5,6,7,8]. Data from a case-control study conducted in multiple Russian regions found that a regular sexual partner who injected drugs was one of most significant risk factors for HIV infection [9].

These data support the case that HIV prevention in Russia needs to target key populations such as PWID. Ample evidence suggests that harm reduction, achieved through sufficient provision of clean syringes and addiction treatment, decreases the risk of HIV transmission [10]. Harm reduction strategies also reduce heroin use, criminal activity and adverse health outcomes, such as death from overdose [11]. Yet, there has been considerable policy resistance in Russia [12] to embrace this pragmatic concept. In contrast, Russian drug policy pursues an approach of punitive policing to reduce drug use and supply [13]. Drug use is illegal in Russia and, according to national legislation, drug dependent people can be arrested and punished based on their drug use alone [14]. Punitive policing practices include extrajudicial arrests for needle possession (which is not illegal in Russia) and police planting of drugs [15]. Such policing strategies might have adverse health implications. Indeed, punitive policing in studies globally has been reported to adversely influence PWID risk behavior by discouraging carrying clean needles, prompting rushed and unsafe injections in riskier environments and keeping PWID from service structures [16].

In several studies in Russia on HIV and health risk, police violence has emerged as a primary theme of human rights violation and impediment to health: in the form of direct physical and legal abuse, but also indirectly through social and societal determinants [17,18]. In a sample of more than 200 Russian PWID interviewed in a landmark qualitative study, informants identified fear of police interference at pharmacies and syringe exchange programs as a primary factor limiting access to clean syringes. Fear of the police also fed reluctance to carry needles and syringes, which deters PWID from using clean syringes, safe disposal of needles, exchange for clean ones, and connection to care [19].

Consistent with the Russian studies, police harassment and discrimination emerged as prominent barriers for PWID to access HIV care in Ukraine [20,21]. In Mexico, police violence and arbitrary detention were reported by PWID to be common, and that police influence was reducing the accessibility of sterile syringes and was driving PWID to hidden locations to inject drugs away from support services [22]. Other ethnographic investigations from the US and Canada posited that police presence and police searches discouraged PWID from carrying clean needles to inject safely [23], prompted ‘rushed’ injections and injecting in riskier environments, discouraged safer injection practices, increased unsafe disposal of syringes, and negatively impacted contact between health services and PWID, as it compromised outreach following the displacement of PWID [24,25,26]. A mixed methods study from India reported that a third of all HIV-positive survey respondents reported recently sharing needles and syringes. Qualitative analysis in this study revealed fear of harassment from police and inadequate access to harm reduction programs were contextual factors that drove these risk behaviors [27].

The relation between policing and health outcomes was independently explored in various studies. In a recent study in St Petersburg, we found that more than half of all PWID surveyed had been arrested by police for possession of needles and syringes (which is not illegal in Russia) or for drugs planted on them. These extrajudicial police arrests were associated with adverse health outcomes such as overdose and risky drug use practices such as needle sharing [15]. Likewise, in a study from Mexico, almost a third (32%) of PWID reported that police involvement led them to rush injections and share needles and syringes, and affected where they bought or used drugs because of the fear that police would interfere with their drug use [28].

One study from the Mexico-US border in female sex workers found that HIV infection was more common among those who had experienced syringe confiscation by police than those who had not, and HIV infection was independently associated with confiscation of syringes by police [29]. In a study from Philadelphia, an increase in street police presence was found to be associated with a decrease in attendance at harm reduction programs, particularly among minorities [30].

The perspective of police officers has been examined previously in various countries, including the US and Russia. Qualitative interviews with police officers in the US found that they blamed PWID for poor life choices and that they feared for their safety when exposed to used needles [31]. A previous examination of police perspectives on injecting drug use and needle and syringe access among PWID in Russia found an uneasy relationship between drug users and law enforcement officers [32]. Police officers were aware of drug users' reluctance to carry injecting equipment linked to their fears of detention or arrest, but perceived PWID primarily as 'potential criminals' and found a 'pre-emptive' approach to the prevention of drug-related crime appropriate, including the official registration of persons suspected or proven to be users of illicit drugs [32].

While the potential adverse substance use and health implications of police involvement have been explored to some extent, less is known about strategies to address the problem in the current societal context in Russia. We therefore sought to explore policing practices in Russia to inform policy making processes related to policing of PWID in Russia, exploring various related perspectives and underlying phenomena. We included PWID and police officers, but probed beyond to gain a variety of perspectives from various other relevant stakeholders such as civil society organizations and international organizations to examine police practices involving Russian PWID.

### Theoretical framework

This was a qualitative study based on a Risk Environment Framework [33,34]. HIV prevention aims to avert the transmission of HIV. As the evidence on the role of genetic, environmental and social determinants on health and risk behavior increases, so does the understanding that HIV transmission is not merely the result of decisions and behavior at the individual level [35]. Rather, the risk of HIV transmission is influenced by the interactions of individual, biological and environmental factors [36].

Environments generate or produce risks and are as important determinants of HIV transmission as are individual behaviors [33]. Behavioral interventions have even been criticized as inadequately “victim blaming”, neglecting to recognize risk and responsibility as being shared between individuals, communities, and environments. In the absence of social and structural interventions and policies [36], behavioral interventions have been found to account for only a modest reduction in HIV incidence.

Risk environment is the space, either social or physical, in which a variety of environmental factors interact and determine the chances of risk occurring [34]. Several types of risk environments (e.g., physical, social, economic, legal, policy) [33] interact at various levels of impact (micro, meso, and macro) [37]. Recognizing that structural violence and vulnerability contribute to the HIV risk environment, in which social and structural factors are inextricably intertwined with dominant political and economic factors, we need an understanding of the structural changes needed within a broad framework of health equity, social justice, and human rights [37] in order to implement effective “structural” HIV prevention [38]. Positive changes in these various types of and at various level of environment to reduce HIV transmission could create an “enabling environment” [37] for HIV risk reduction.

Both risk environment and individual behavior are modifiable and influence each other [36]. This implies that effective, comprehensive HIV prevention not only comprises interventions targeting individual behavior, but also modifications to local environments conducive to and supportive of health promotion and behavior changes. We therefore applied the Risk Environment Framework seeking to understand the dynamic and complex relationships of individual-environment interactions in the production and reduction of risk. Related to our study question, we applied the framework to explore empirical evidence on the complex phenomenon of police violence against PWID, integrating a variety of perspectives in the Russian country context.

## Methods

### Procedures

We collected data through key informant interviews with a broad spectrum of experts to explore strategies to address the risk environment of Russian PWID. We conducted 23 in-depth interviews (IDI) with study participants who had knowledge of drug-related public health policy making in Russia.

### Sampling

We purposively sampled prospective study participants. With the help of Russian and international civil society organizations (CSOs) in Russia, we recruited key informants deemed information-rich and reflecting a spectrum of perspectives. We approached PWID, police officers, addiction care providers, NGO representatives, and experts from international organizations in Russia through our network of contacts at CSOs and addiction clinics at the study sites. While drug policy in Russia is made on a federal level in Moscow, we aimed to sample participants at various sites to explore data across different sites with a spectrum of policy and political backgrounds on policing, drug risk and HIV issues. Interviews were conducted at the following sites: Moscow, Russia’s capital and largest city, with 11.5 million inhabitants and 52,000 PWID registered with the authorities (out of an estimated 150,000) [39]; St. Petersburg, a city of 5 million, one of Russia’s urban areas most affected by opioid addiction with 57,000 registered PWID and an estimated PWID population of over 80,000 [40]; and a provincial city in the North Caucasus in the South of the Russian Federation, home to several thousand PWID [39].

In total, from March through August 2012, we collected data from 23 participants, including 6 PWID and 3 police officers from Moscow or the North Caucasus, 3 Russian addiction physicians (narcologists) and one from another former Soviet country, 4 representatives of Russian CSOs in Moscow or St. Petersburg and one from a CSO in another country, as well as 5 experts from international NGOs or international organizations in Russia. To gain a diverse range of contextual perspectives, we included key informants (n = 4) from other former Soviet Republics who were familiar with Russian drug policy. Individuals who were knowledgeable about drug use and drug policy in Russia were eligible. Of 32 individuals approached for participation, 9 (28%) declined or withdrew their consent, mostly police officers and health care providers.

### Data Collection

Interviews were conducted by KL and FL, a male and female researcher, respectively, who are familiar with the Russian health and addiction treatment systems, fluent in both English and Russian, have a medical and anthropological background, and are trained and experienced in qualitative methodologies. Based on pilot-tested, semi-structured interview guide with identical questions for all participants, we used progressive focusing (i.e., further modifying and adding questions over the course of the study in an iterative process) to explore and reflect on emerging findings and topics.

Following oral informed consent based on a consent script, we conducted the interviews in a private location that was convenient for participants or over the phone, and audio-recorded and transcribed them verbatim. Interviews lasted between 36 and 102 minutes. A native bilingual Russian-English speaker translated the interviews conducted in the Russian language into English during transcription from audio files for analysis.

During data collection and analysis, we ensured that participants remained anonymous. We obtained oral rather than written consent to further reduce the risk of breach of participants’ identities through written consent documents, particularly in view of the vulnerability of PWID and this study’s sensitive topic. We recorded oral consent for study participation in a de-identified spreadsheet assigning participants’ consecutive numbers.

The study, including its consent procedure, was approved by the Institutional Review Board of Boston Medical Center (H-31511).

### Data Analysis

We used Nvivo software [41] to code qualitative data. We used a thematic analysis approach [42] to inductively generate new insight into the phenomenon of police involvement and potential strategies to address it. Two authors of the study (KL, FL) who were also the interviewers, coded all data. We started the first, open coding cycle by formulating units of organization and analytic codes [43]. We then compared and refined first-cycle codes, identified higher-level codes and relationships among codes, and agreed to the codes to include in the code list based on consensus among both coders. The second coding cycle involved a final stage coding of all interviews using a final and full set of emergent themes. We recoded and re-categorized data to identify recurrent themes and patterns [44] as presented in the results section.

We use the COREQ [45] and RATS [46] guidelines to report our study.

## Results

### a) Forms of police abuse

Data from the 23 in-depth interviews revealed physical violence and other human rights abuses, such as beating, arrests, verbal harassment, and coercion. In the interviews, arbitrary police arrests emerged as a common experience among PWID. Reported abusive policing practices include physical violence as a ubiquitous phenomenon in Russia:

“I don't know of one single drug user who has not been beaten up by police.”

PWID 1 North Caucasus

Many respondents emphasized that high stigma against drug users is persistent, and that violence is often perceived as normal when perpetrated against drug users. A CSO representative summarizes:

“Regular routine violations by police, such as illegal detention of people, planting drugs, beating drug users, extorting money from people to avoid being arrested, bribes, or raping of drug users, sex workers, and things like that. This kind of behavior towards drug users is quite normal for the police. And also, for drug users, themselves, it’s perceived as quite normal behavior coming from the police.”

Russian CSO staff 2, Moscow

### b) Consequences, substance use implications of police abuse

Police violence, fear from police prosecution and the resulting lack of trust in existing laws and their enforcement deter drug users from seeking health care or other services. This theme emerged from the accounts of various key informants, as this CSO representative describes:

“Drug users just hide from the state in any form. They don’t go to medical services, because they’re afraid. And they sometimes don’t go to pharmacies, because they are afraid to be confronted with the police. And they are so stigmatized and scared, that they don’t want to have any interaction with any official structure, or service. So police just scare people and make people take risky decisions.”

Russian CSO staff 2, Moscow

Another CSO representative argued that police involvement distracts drug users further from harm reduction behaviors:

“I mean, HIV’s no priority for drug users. They don’t think about HIV. They think how not to get caught by police, how to save their money from the police, how not to get beaten up by the police. You know? They don’t think, should I take THIS syringe or THAT syringe? How do I protect myself from HIV? These are not the kind of decisions people take on a daily basis. These are not the risks they perceive. Their risks are police and their violence, and the risk to get into prison.”

Russian CSO staff 1, Moscow

Most respondents think that arrests do not deter drug use, as this drug user reiterates:

“Drug users need money. So what do they do? They steal, and police should prevent them from doing that. [*What should police do to deter drug use*?] Their arrests do not deter drug use. [*How can police deter drug use*?] I think that putting drug users in jail is not the answer. Drug users are sick persons. Here in Russia, jails are full of drug users. […] I think in Russia drug users should not be jailed. They should receive therapy. “

PWID 1 North Caucasus

### c) Police abuse as risk environment for PWID in Russia

PWID are highly stigmatized in Russia and often not perceived as humans, and this attitude seems to facilitate violence against PWID. As this police officer summarizes colleagues' attitude:

“For police officers, the attitude towards people who use drugs is to fight their crimes. And in the eyes of police officers, people who use drugs are basically not human beings. They’re animals who are hooked up to a needle. And for that needle, they are capable of killing their own mother. And so that’s why they don’t deserve to be in the free, open society. You know what is interesting? Human rights—the key word is human. Human rights are for humans. But drug users are not human. Therefore, they don’t have human rights.”

Russian police officer 2 Moscow

Through the dehumanization of PWID, rather than perceiving them as individual human beings and patients with a chronic disease, drug dependent people are equated with drug-related crime. A Russian scholar sees this as being at the core of violence against drug users:

“The violence against addicts is a reflection of the relation to them not as patients, but as immoral and criminal persons.”

Russian addiction physician 3

Others, as this police officer, are more understanding of the issues PWID are confronted with, but generally maintain the belief that they should be dealt with as a criminal justice matter:

“I now have a more clear understanding of what it means to be dependent on drugs. He doesn’t belong to himself, he really is dependent. It’s necessary to catch and jail a drug user, to isolate him from society. This is because to make money for drugs, he steals and engages in criminal behavior.”

Russian police officer 1 North Caucasus

For many, police abuse seems difficult to address, as abusive practices allow for additional sources of income for some officers. Violence is used to enforce extortion of money from PWID in order for them to avoid arrests or drug crime accusations:

“A drug user is a source of income for police. […] I had to provide money to avoid being put in jail. If I hadn’t paid, I would have been put in jail. Even though those weren’t my drugs that were on me, they simply put them in my pocket, just because I was a drug user. They have a quota to fill.”

PWID 3 North Caucasus

“For police, beating a drug user is an easy way to get some cash… sometimes small, sometimes big money, it depends. Even if the person didn’t commit a violation, then the police officer can say anything, like, ‘I know you’re a junkie. If you don’t give me the money I’ll just plant some drugs on you and you will be imprisoned for several years,’ and so anyone would just give them the money.”

Russian CSO staff 1, Moscow

Many drug users feel profoundly helpless vis à vis the arbitrariness of police, who act with impunity. Police can take PWID into custody at any time solely based on their drug use, and then proceed at their discretion. As one CSO worker accounts in the following:

“If you are a drug user and you fall in the hands of police, you’re at their mercy, because they can do with you whatever they want. There’s no one who’s going to protect you, there’s likely no one who’s going to believe you if you talk about abuse. […] I mean, the police needs to be held be accountable for their action. But now, the system is so corrupt that many criminal actions *[by the police]* remain unpunished, because the system is protecting itself”

Russian CSO staff 2, Moscow

### d) Areas of opportunity to inform policy: approaches to mitigate police violence

All key informants contributed thoughts and ideas on potential approaches to mitigate the phenomenon of police violence and its public health implications in Russia. Most suggestions were directed at the following areas:

### 1) Strategic police education in public health aspects of drug use

Representatives from CSOs serving PWID and international stakeholders suggested that training of police officers to gain an understanding of drug addiction as a chronic disease might facilitate linking PWID to care as an alternative to sanctioning them:

“The role of police should be informed much more by public health principles. Police need to be trained on health questions, so that instead of chasing after people who are buying a dose of heroin for personal use, essentially chasing after people who have an illness that needs to be treated rather than punished, instead they need to play much more of a role facilitating entry of people into treatment.”

International CSO staff 2

PWID, CSOs and international organizations all perceived police education and training as a possible way of improving the interaction between police and drug users:

“Over the years there have been some trainings. In Kazan, for example, they trained police. After the training, the police understood much more the purpose of the harm reduction sites, and how they served a public health interest. They understood that this was not a place where people were selling drugs.

International CSO staff 1

Others are more skeptical about the potential of police education alone. Recent experiences suggest that the Russian political system is not conducive to change in attitudes towards drug users. This expert expresses lack of faith in system change, in spite of trusting that individuals may be amenable:

“*[You said that educating police would not bring much if there wasn’t a culture change in the relation between drug users and police*.*]* I mean, maybe one or two police officers, okay, they will beat people less strongly next time. But on a system level, it will not change. Educating the police is a nonsense intervention. *[Why*?*]* Because it doesn’t help. You cannot teach someone to fly, if the system is such that it pulls him back to the ground. First, you need to address corruption and have accountability in the police. No, you cannot just educate someone not to be violent.”

Russian CSO staff 2, Moscow

### 2) Aligning shared goals of public safety and public health

Several drug users and CSO representatives expressed visions that reach beyond the current adversarial police-drug user relationship. They advocated for the public safety and public health sectors to pursue similar goals, thus fostering an understanding between police officers and drug users that might allow drug users to improve their self-efficacy:

“Police can serve as a bridge to medical services or harm reduction services, for drug users to take them to low-threshold services. […] In many other countries it happens like this. Police, who face most of all bad things that happen because of drugs and drug criminality, can advocate for access to health services for drug users and often for drug policy.”

Russian CSO staff 2, Moscow

**Fig 1:**
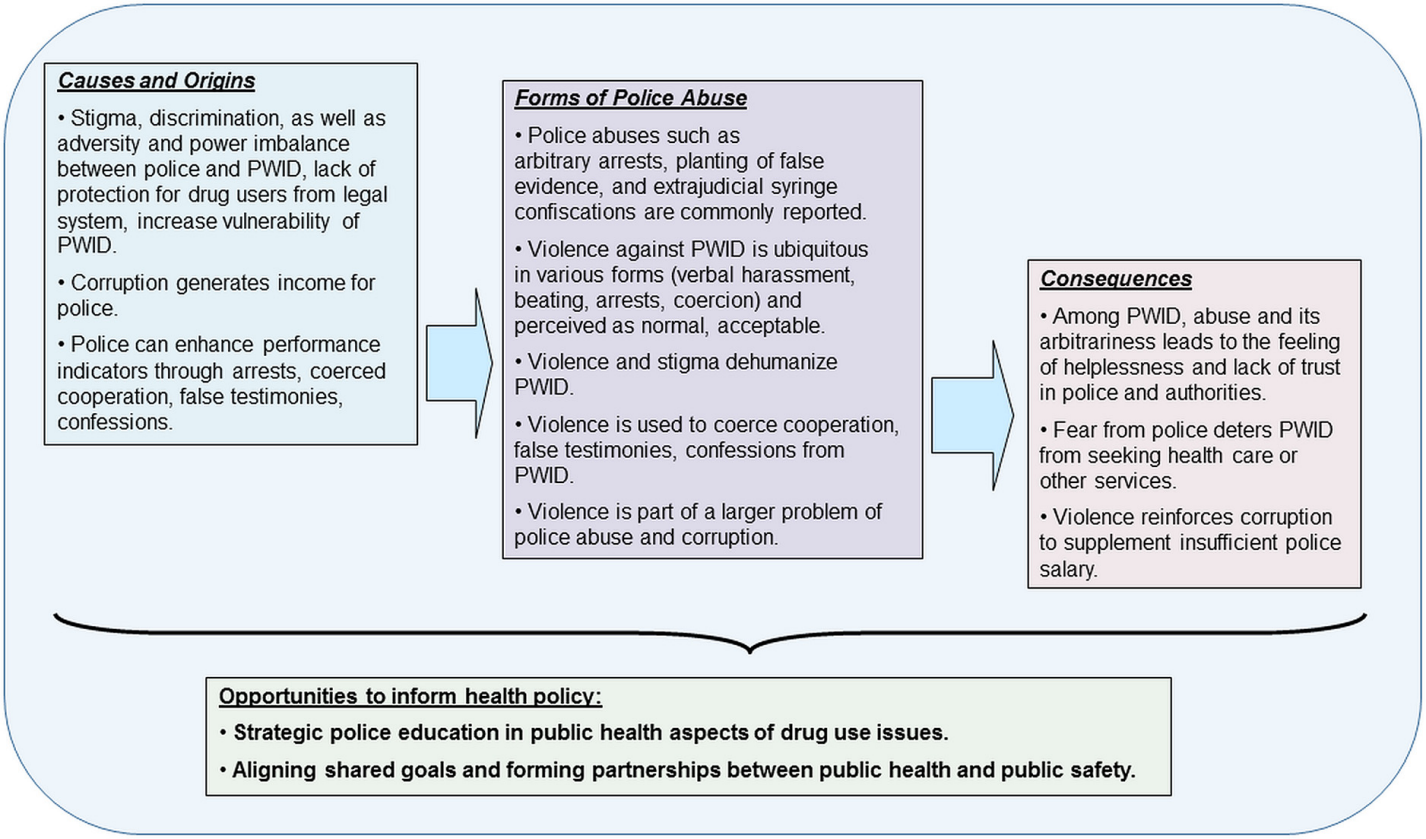
Expansion of topics emerging from quotes in key informant interviews. Themes and concepts characterize police involvement as an element of risk environment of Russian PWID, and offer attitudes and insights into possible policy approaches to address structural risks in Russia.

Further findings on causes and forms of police abuse, as well as health consequences and opportunities to inform health policy are summarized in Fig 1.

## Discussion

Punitive policing practices contribute to the risk environment of Russian PWID. This study describes the phenomenon of police involvement with PWID at different study sites, indicating that the reported abusive police involvement might not be a localized phenomenon in Russia. Our exploration among various key informants facilitated an understanding of forms, causes and consequences of police practices and identified related public health policy opportunities in Russia. Importantly, this study aimed to identify opportunities to use empiric evidence to address the problem of police violence and inform effective policy (Fig 1).

### Characterization of policing practices

This study’s data suggest that police involvement with PWID in Russia is often marked by violence and human rights abuses, which are facilitated by the high stigma against both substance use and HIV infection. Stigma is the dehumanization of the individual based on their social identity or participation in a negative or an undesirable social category [47]. In this study’s context, stigma refers to the societal and political exclusion of PWID as a consequence of their perception in Russian society as socially not productive individuals. This leads to a discrimination of PWID in Russia on the grounds of structural vulnerability and structural violence: The accounts of police violence in our study and the related health implications emphasize how structural violence and vulnerability contribute to the HIV risk environment, in which social and structural factors are inextricably intertwined with policy and law enforcement factors.

Russian studies on HIV and health risk confirm our findings of police violence as a human rights violation and impediment to health. Extrajudicial policing practices, such as unjust arrests, planting of false evidence, and the extortion of money or drugs, were commonly reported by PWID in another qualitative Russian study, which described various forms of police violence, such as torture and physical violence, even rape, as an act of "moral" punishment and to coerce confessions [18]. In a Russian study of police officers, they viewed PWID primarily as “potential criminals”, whose 'pre-emptive' detention might prevent drug-related crime [32].

Adding to existing research, our exploration sampled stakeholders beyond PWID and police officers. This study also included representatives from CSOs in Russia and other former Soviet republics, international organizations in Russia, and Russian health care providers. We found that in Russia, other systemic factors, such as police officers’ insufficient income from salary or inadequate opportunities for PWID to obtain legal protection, fuel police abuse mechanisms (Fig 1) and thus reinforce structural vulnerability and structural violence against PWID [33].

Our findings further suggest that abuse and violence are rooted in the high societal stigma associated with HIV infection and drug use that exists in Russia [48], which leaves PWID particularly prone to infractions of their rights [49]. A systematic review emphasized the link between human rights violations against PWID and an increased vulnerability to HIV infection and worse access to HIV and addiction services [50]. Common human rights abuses of PWID include not only discriminatory access to HIV and addiction therapy or denial of harm reduction services, but also abusive law enforcement practices [51,52,53,54,55].

As previously postulated [52], our results confirm that police involvement and fear of police are prominent structural factors adversely impacting not only health care seeking, but also risk behaviors: police violence and human rights violations have been repeatedly shown to be associated with adverse substance use risks and health outcomes in various settings. Although, as is the case in Russia, syringe possession was legal in these locations, police arrests and confiscation of drugs and syringes were associated with receptive needle sharing in analyses from Vancouver, Canada [53] and Mexico’s border region with the USA [54]. A human rights investigation from India found an association between police arrests of male PWID, being threatened with violence or actual physical abuse and suicidal ideation [51]. In a study of experiences of PWID in Bangkok, Thailand, the planting of drugs as false evidence was commonly reported and associated with non-fatal overdose and syringe-sharing [55]. In a qualitative study there, which reported human rights violations and corruption similar to this study’s findings in Russia, participants were reluctant to report abuses to the authorities, given the limitations of the justice system [56]. It has consequently been argued that much of the needed structural HIV prevention advances are political in nature [37], calling for structural changes within a broad framework of health equity, social justice, and human rights [57].

### Strategies for drug policy

As essential elements of this study, we explored among study participants how to strategically use empiric evidence in the context of considerable policy resistance in Russia. Respondents remarked that current policing practices are not capable of controlling the problem of drug use in Russia. They suggested that training of police in health issues of drug users was an essential part of aligning public safety and public health strategies. Economic and political interests in maintaining repressive drug policing have been widely recognized as undermining attempts to change drug legislation and their enforcement in Eastern European countries [58]. For police, drug users are perceived as a “huge resource” of money, performance statistics, and “operational information”, which disincentivizes policing change [58].

Changes in various aspects of the environment can influence HIV risk [38]. Programs and policies that change the environments in which risk behavior occurs may either facilitate behaviors that reduce HIV transmission risks, or may discourage engaging in risk behavior by making it more inconvenient. Thus, structural prevention interventions addressing police behavior towards PWID might impact HIV transmission in Russia. In addition to addressing the overarching issue of corruption that emerged in our data, effective police trainings on harm reduction to facilitate an enabling environment for HIV prevention need careful implementation strategies, emphasizing commonalities of the police public safety mission and public health goals. Both sectors should seek to reduce unnecessary detention, incarceration, and HIV transmission risk associated with being in prisons and jails [59]. As previous evaluations have suggested, police trainings might be one catalyzing element as part of broader efforts to align police and public health goals [31,60,61,62]. Police abuse represents a risk for law enforcement officers, e.g., when they handle potentially contaminated needles. Police education on harm reduction can emphasize the benefit for occupational safety risk (e.g., a lower needle stick risk secondary to needle exchange activities) and provide a platform of common understanding [63]. Current evidence suggests that even brief police trainings may lead to a better understanding of harm reduction principles, help align police work with public health efforts [64] and build understanding and harmony between the two sectors [65]. Seattle offers an example, where officers at the time of arrest can decide whether to divert PWID to addiction treatment [66].

Creating shared measures of success could foster synergy between public safety and public health goals. This is also means to frame harm reduction principles with respect to the operational environment of law enforcement. A suggestion for such a strategy comes from police officers in a country with restrictive drug policies and strict enforcement practices. In a Malaysian study, police recommended a change of their key performance indicators from "persons arrested" to "persons arrested referred to treatment" [67]. Thus, public health organizations could translate the police outcome indicator “arrest” into the healthcare output indicator “PWID connected to care”. The effect of such policies in Russia would have to be evaluated with the caveat of the government’s opposition to internationally recognized harm reduction measures and its proscribing of opioid agonist therapies with methadone or buprenorphine [12].

Both risk environment and individual behaviors are modifiable and influence each other. This implies that effective, comprehensive HIV prevention not only comprises interventions targeting individual behavior, but also includes modifications to local environments conducive to and supportive of health promotion and positive behavior changes. In the case of police involvement with PWID, this requires the public safety sector’s understanding of the risk environment of PWID and public health sector’s understanding of police operational environments. Since protecting the human rights of PWID is essential to improving their health, rights-based structural interventions could create an enabling environment, which addresses both individual-level risk factors (e.g., injection and sex practices) and structural risk factors (e.g., policing and drug law enforcement).

### Limitations

This study is limited in sample size with data from multiple sites and several categories of informants. This limits the generalizability of our findings beyond the informants and settings included in our study. We tried to address this limitation by sampling three additional PWID study participants (beyond the three planned) until we achieved saturation (i.e., no new themes emerged during analysis).

We sampled primarily proponents of progressive drug policy and sampled few opponents of policy change, as they were less amenable to be included in our study. This limits our ability to more closely investigate areas of resistance to change.

## Conclusions

Limiting the HIV epidemic in Russia requires not only treatment of affected people and prevention programs to modify individual behaviors among key populations such as PWID, but importantly also needs drug enforcement policies informed by the best available empiric evidence and non-abusive policing practices. Police trainings and introduction of a key police performance indicator such as the referral of PWID to addiction or HIV treatment are two possible interventions to modify the risk environment of PWID in Russia while considering law enforcement’s operational environment. Addressing the risk environment by implementing evidence-informed drug and HIV prevention and treatment programs that harmonize public health and public safety might prevent further spread of HIV in Russia.
